# Prognostic risk factors for lymph node involvement in patients with endometrial cancer

**DOI:** 10.4274/tjod.52385

**Published:** 2017-03-15

**Authors:** Tayfun Toptaş, Tayup Şimşek, Şeyda Karaveli

**Affiliations:** 1 Antalya Training and Research Hospital, Clinic of Gynecologic Oncological Surgery, Antalya, Turkey; 2 Akdeniz University Faculty of Medicine, Department of Gynecologic Oncological Surgery, Antalya, Turkey; 3 Akdeniz University Faculty of Medicine, Department of Gynecopathology, Antalya, Turkey

**Keywords:** Endometrium cancer, lymph node dissection, risk factors

## Abstract

**Objective:**

We aimed to analyze variables affecting lymph node (LN) involvement and to assess the need for systematic lymphadenectomy in patients with endometrial cancer (EC).

**Materials and Methods:**

A single centre retrospective analysis was conducted in a total of 128 consecutive patients with EC who underwent systematic pelvic or combined pelvic and paraaortic lymphadenectomy between 2009 and 2012. Mann-Whitney U, chi-square, and Fisher’s exact test were used for univariate analyses when appropriate. Variables with a p value <0.05 in the univariate analysis were included into a multivariate logistic regression analysis. The effects of variables on LN involvement are reported using adjusted odds ratios (ORs) and 95% confidence intervals (CI).

**Results:**

In univariate analysis, grade 2-3, tumor size ≥3 cm, deep (≥50%) myometrial invasion, presence of cervical, adnexal or omental involvement, positive peritoneal cytology, open surgical approach (laparotomy), combined pelvic and paraaortic lymphadenectomy and number of total LNs removed (>30) were found associated with LN involvement. However, the number of total LNs removed (>30) was the only independent variable that predict LN involvement in multivariate analysis [OR: 15.08; 95% CI: (1.28-177.59); p=0.03].

**Conclusion:**

This study demonstrates that the more LNs removed during staging of EC, the greater the probability of finding LN metastasis.

## INTRODUCTION

Endometrial cancer (EC) is the most common gynecologic malignancy in developed countries. Its age-adjusted incidence is increasing, probably due to increased life expectancy and obesity. However, the mortality rate has increased more rapidly than the incidence over the past three decades^(1)^. One explanation for this discrepancy is that patients are being diagnosed at an older age, which leads to an increased rate of high-risk histologies and advanced-stage cancers.

EC is staged surgically based on the International Federation of Gynecology and Obstetrics (FIGO) 2009 staging system^(2)^. Lymph node (LN) metastasis is one of the most important prognostic factors for EC^(3)^. Although a systematic lymphadenectomy is an essential part of staging surgery, FIGO did not define the optimal limits for lymphadenectomies, nor the adequate number of LNs required for the comprehensiveness of the procedure. On the other hand, it is well known that lymphadenectomy may be associated with increased complications, mainly including lymphedema, vascular, ureteral and visceral injuries, deep vein thrombosis, chylous ascites, and ileus^(4)^.

In the current study, we aimed to analyze variables affecting LN involvement and to assess the need for systematic lymphadenectomy in patients with EC.

## MATERIALS AND METHODS

A single centre retrospective analysis was conducted in a total of 128 consecutive patients with EC who underwent systematic pelvic or combined pelvic and paraaortic lymphadenectomy between 2009 and 2012. Patients were excluded if they had primary synchronous malignancy or if they had no LN dissection. Clinicopathologic data including age, type of surgical procedure, tumor histotype, tumor size, grade, depth of myometrial invasion, lymphovascular space involvement (LVSI), cervical involvement, adnexal involvement, positive peritoneal cytology, number of LNs, and LN involvement were extracted from patient charts and the institutional database following approval of institutional review board of Akdeniz University. Written informed consent was not required for this type of retrospective study. This study has been approved by the Local Ethics Committee of the Akdeniz University (date and approval number: 2012/1205). The study was performed in accordance with the ethical standards described in an appropriate version of the 1975 Declaration of Helsinki, as revised in 2013.

As a routine policy of our institution, patients with newly diagnosed EC were offered treatment with total hysterectomy and bilateral salpingo-oophorectomy with systematic pelvic lymphadenectomy. Paraaortic LN dissection was added to pelvic LN dissection in patients with at least one of the following risk factors: a) non-endometrioid histology, b) grade 2 or 3 endometrioid adenocarcinoma, c) deep (≥50%) myometrial invasion on intraoperative frozen-section examination.

The primary endpoint of the study was determination of independent factors influencing LN metastasis. The Stata software package was used for statistical analyses (Special Edition v11.2 for Macintosh OSX, StataCorp, Texas, USA). Mann-Whitney U, chi-square, and Fisher’s exact tests were used for univariate analyses when appropriate. Variables with a p value <0.05 in the univariate analysis were included into a multivariate logistic regression analysis. The effects of variables on LN involvement are reported using adjusted odds ratios (ORs) and 95% confidential intervals (95% CI).

## RESULTS

The mean age at surgery was 59.3±11.2 years and the majority of patients (86.7%) had open surgery. Sixty-six patients (51.6%) had pelvic lymphadenectomy alone, and 62 (48.4%) had combined pelvic and paraaortic lymphadenectomy. The median number of pelvic LNs removed, paraaortic LNs removed, and total LNs removed (both pelvic and paraaortic) were 24, 15, and 32, respectively. Most patients had endometrioid histology (75%). LN involvement was detected in 17.9% of the patients, deep myometrial invasion in 45.3%, LVSI in 25%, cervical involvement in 16.4%, adnexal involvement in 11.7%, omental involvement in 4.7%, and positive peritoneal cytology in 8.6% (Table 1).

**Figure 1:**
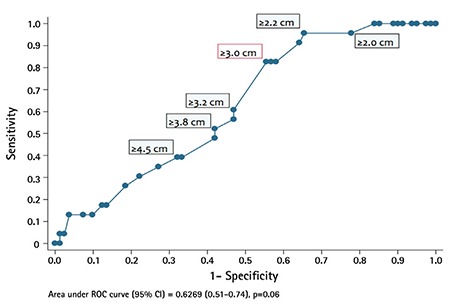
Receiver operating characteristic analysis for calculating cut-off value of tumor size in predicting lymph node metastasis *ROC: Receiver operating characteristic, CI: Confidential interval*

In the univariate analysis, grade 2-3, tumor size, deep (≥50%) myometrial invasion, presence of cervical, adnexal or omental involvement, positive peritoneal cytology, surgical approach (laparotomy vs. laparoscopy), combined pelvic and paraaortic lymphadenectomy, and the total number of LNs removed were found associated with LN involvement (Table 2). A receiver operating characteristic analysis was performed to determine the tumor size that would be the most significant in predicting LN involvement (Figure 1). The cut-off value was found as 3 cm with an area under the curve of 0.626 [CI: (0.51-0.74); p=0.06].

However, in the multivariate analysis, the total number of LNs removed (>30) remained as the only independent variable that predicted LN involvement after adjustment for other confounders [OR: 15.08; 95% CI: (1.28-177.59); p=0.03] (Table 2).

## DISCUSSION

The current study examined factors influencing LN involvement in patients with EC. Our results identified the total number of LNs removed as the only independent predictor of LN metastasis; this finding emphasizes that as many LNs as possible should be removed irrespective of preoperative tumor characteristics in order to determine LN metastasis.

Defining the role and extent of lymphadenectomy is one of the main controversies in the management of patients with EC. Lymphadenectomy provides pathologic and prognostic data, determines the exact extent of disease, and need for adjuvant therapy. It may also have a potential therapeutic effect in patients, particularly with extrauterine disease^(5,6,7)^.

Overall LN metastasis in patients with EC has been reported to range from <1% to 34%, according to tumor grade, histotype, and depth of myometrial invasion^(3)^. It is widely accepted that in a subset of patients (low-risk group) with low-grade endometrioid histotype, small tumor size (<2 cm) and no deep myoinvasion, lymphadenectomy may be omitted without a negative impact on prognosis^(8)^. This group of patients has a relatively small risk (1-3%) for lymphatic dissemination^(3)^. However, it is difficult to identify these low-risk patients preoperatively because of variability in tumor grade and depth of myoinvasion on final histopathology^(9,10)^. Therefore, the true risk may be greater than that estimated. Although two randomized controlled trials (RCTs) reported that lymphadenectomy did not improve the outcomes of patients, there are some critical issues with regard to these RCTs including adjuvant therapies, number of LNs removed, and extent of lymphadenectomies^(11,12)^. Radiotherapy was given to an equal number of patients in each treatment arm, which led to overtreatment of non-lymphadenectomy groups.

Sentinel LN biopsy can represent a compromise between no lymphadenectomy (leaving a small risk for LN metastasis) and full lymphadenectomy (adding a potentially morbid procedure for a significant part of the patients). It improves detection of LN metastases by allowing detection of micrometastases using ultrastaging (serial sectioning) of target LNs. In a multicenter study of 304 women with presumed low- or intermediate-risk disease, sentinel LN biopsy and ultrastaging detected metastatic LNs in three-fold greater than standard lymphadenectomy (16% vs. 5%)^(13)^. However, the implications and management of micrometastases or isolated tumor cells detected through ultrastaging are not yet clear. No prospective RCTs have compared outcomes of disease between patients who underwent sentinel LN biopsy and those who received systematic LND. In addition, risk of non-sentinel LN positivity (false negativity), which has been reported as approximately 5%, is a potential handicap for sentinel LN biopsy^(14)^.

Today, systematic pelvic lymphadenectomy is still the safest way to detect LN metastasis in patients with EC who have low-risk features. It allows elimination of LN metastasis in approximately 99% of patients. Potentially missed cases are patients with isolated paraaortic LN metastasis^(15)^. Combined pelvic and paraaortic lymphadenectomy may be reserved for selected patients with high-risk features^(16)^.

**Study Limitations**

As with all studies, the results of this study are not without limitations. Retrospective single center studies, such as the current one, are inherently susceptible to selection and referral bias. On the other hand, the main strengths of our study include the detailed analyses of various clinicopathologic factors that may have an impact on LN metastasis, and performance of uniform staging surgeries using a consistent surgical policy by subspecialized gynecologic oncologists.

## CONCLUSION

In conclusion, the current study demonstrates that the more LNs removed during staging of EC, the greater the probability of finding LN metastasis. Following clarification of the most appropriate adjuvant therapy regimens for sentinel LN biopsy procedures in pending trials, the role and therapeutic effect of lymphadenectomy may be assessed more effectively.

## Figures and Tables

**Table 1:**
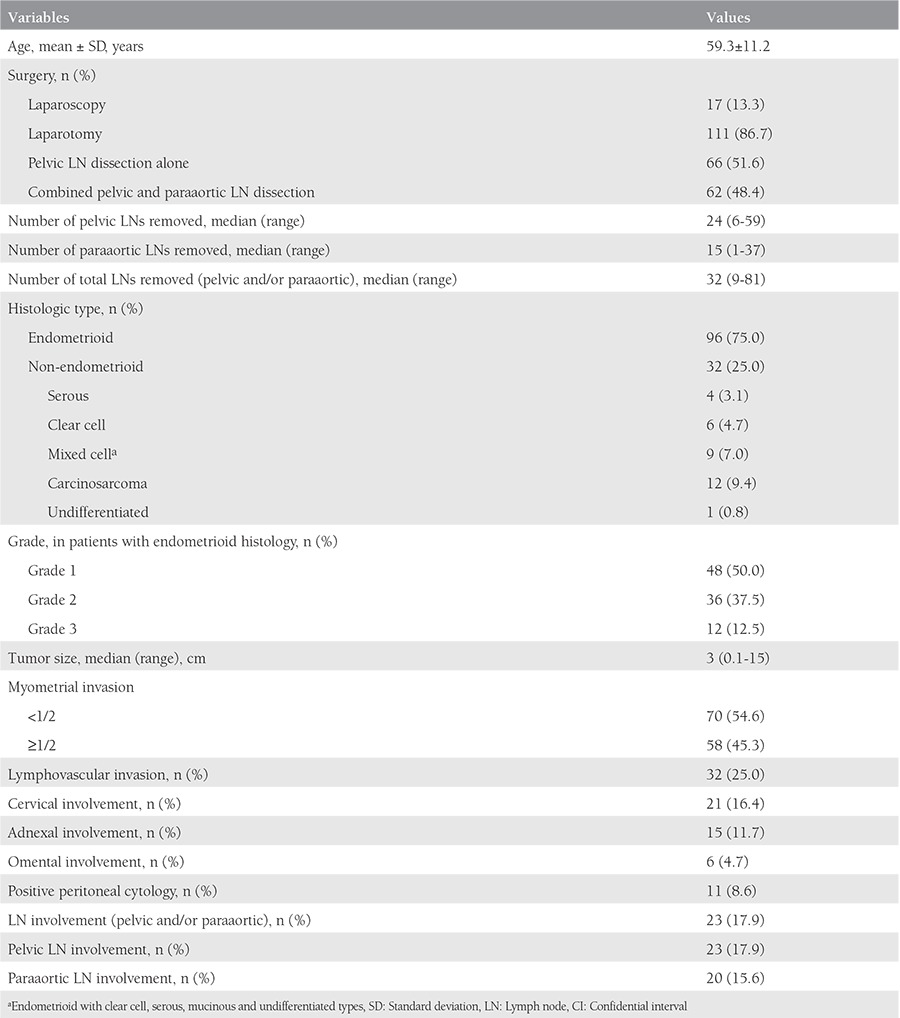
Clinical and pathologic characteristics of patients

**Table 2:**
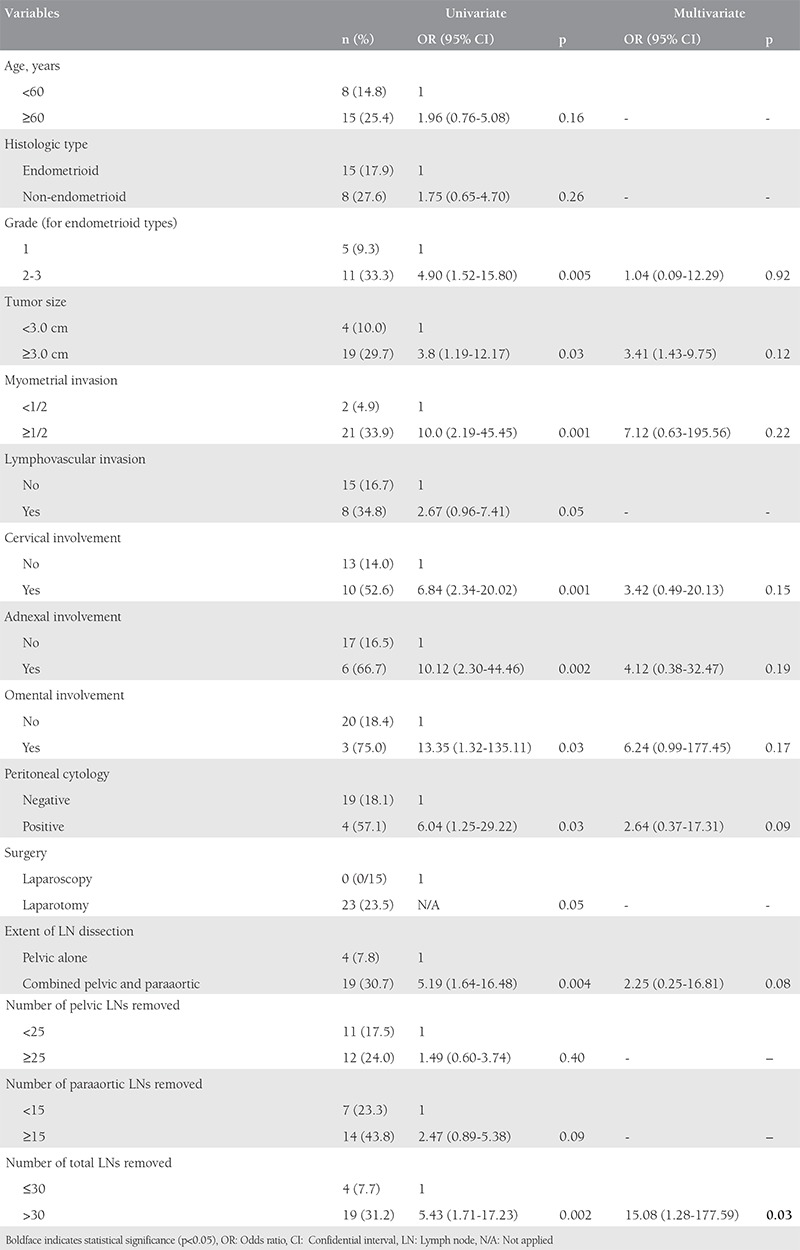
Univariate and multivariate logistic regression analysis of factors predicting lymph node metastasis
